# Further integrating social context into comparative and environmental physiology

**DOI:** 10.1242/jeb.251374

**Published:** 2026-02-09

**Authors:** Shaun S. Killen, Daphne Cortese, Lucy Cotgrove, Emmanuelle Chrétien, Emil Christensen, Amélie Crespel, Jolle Jolles, Mar Pineda, Izzy C. Tiddy, Cheng Fu, Daiani Kochhann, David J. McKenzie, Amelia Munson

**Affiliations:** ^1^ School of Biodiversity, One Health & Veterinary Medicine, College of Medical, Veterinary & Life Sciences, University of Glasgow, Glasgow, G12 8QQ, UK; ^2^ UMR Marbec, Université de Montpellier, Ifremer, CNRS, IRD, INRAE, Montpellier, France; ^3^ Natural Resources Institute Finland (Luke), Paavo Havaksen tie 3, 90570 Oulu, Finland; ^4^ Department of Biology, Chemistry and Geography, University of Quebec in Rimouski, Rimouski, Canada, G5L 3A1; ^5^ Department of Biology, Turku University, 20500 Turku, Finland; ^6^ Department of Ecology & Complexity, Center for Advanced Studies Blanes (CEAB-CSIC), Blanes, Girona – 17300, Spain; ^7^ Laboratory of Evolutionary Physiology and Behavior, Chongqing Key Laboratory of Conservation and Utilization of Freshwater Fishes, Chongqing Normal University, Chongqing, 401331, China; ^8^ Center of Agrarian and Biological Sciences, Acaraú Valley State University, Sobral 62010-295, Ceará, Brazil; ^9^ Department of Wildlife, Fish and Environmental Studies, Swedish University of Agricultural Sciences, 901 83 Umeå, Sweden

**Keywords:** Physiological plasticity, Social stress, Environmental tolerance, Indirect genetic effects, Experimental design, Metabolic regulation

## Abstract

Environmental factors such as temperature and oxygen are well-established modulators of animal physiology, but the influence of social context remains under-integrated into comparative and environmental physiology. Although numerous studies across behavioural, ecological and biomedical fields show that social interactions alter metabolic, hormonal, immune and stress-related traits, these insights are not routinely incorporated into physiological study design or interpretation. Social effects arise through mechanisms such as isolation, dominance hierarchies, altered energy use and social buffering, and can amplify or dampen responses to abiotic stressors. Because metabolic and hormonal pathways regulate multiple physiological systems, socially induced shifts can cascade to affect cardiovascular, immune, neural, digestive, osmoregulatory and reproductive function over both acute and evolutionary time scales. Thus, overlooking social context places researchers at risk of taking two critical missteps in comparative and environmental physiology: (1) measuring animals under socially unrealistic or uncontrolled conditions, which can yield unrepresentative physiological estimates; and (2) extrapolating these findings to natural populations where trait expression is influenced by social dynamics that are absent from the experimental context. Together, these issues might bias estimates of physiological trait values, plasticity and heritability, and limit the ecological relevance and predictive power of physiological research. Here, we outline general strategies to incorporate social context into experimental design, including the use of emerging tools that allow physiological measurements in naturalistic social settings. Integration of social context, alongside abiotic drivers, will improve our capacity to predict organismal responses to environmental change through comparative physiological research.

## Teaser

**Summary:** Social environments affect all aspects of animal physiology. We highlight how overlooking social effects might lead to inaccurate physiology-based predictions of species’ responses to environmental change.

## Introduction

The physiology of individual species has been shaped over evolutionary time in response to numerous factors, both abiotic (e.g. temperature and oxygen availability; Schmidt-Nielsen, 1997) and biotic (e.g. predator presence and food availability; McCue, 2010, Clinchy et al., 2013; Rosenfeld et al., 2015), but also shows phenotypic plasticity (see Glossary) in the face of short-term and within-generational environmental conditions. The field of comparative physiology examines these interactions between an animal's environment and its physiology, with an increasing focus on how physiology underlies the ability of populations and species to cope with environmental change (Seebacher et al., 2023).

**Fig. 1.**
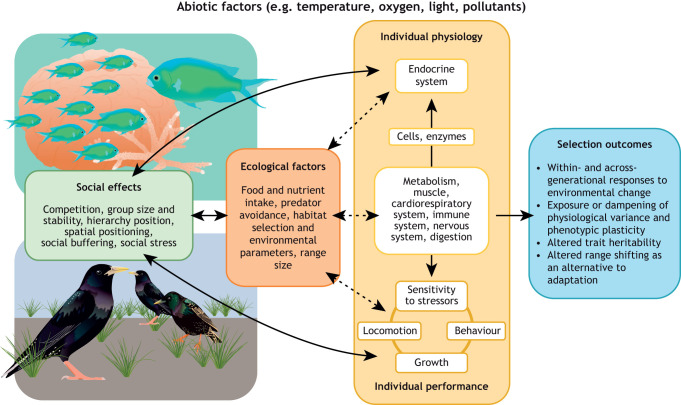
**Socially mediated physiological cascade.** The social environment can affect individual physiology and evolutionary responses to environmental change through numerous pathways. Social effects (e.g. social stress or buffering; green box) can modulate the primary stress response and have subsequent effects on tissue, organ or whole-animal physiology and performance via a socially mediated physiological cascade (yellow box). Social factors such as spatial positioning within groups can also directly affect locomotor costs and other aspects of behaviour (e.g. spontaneous activity) which can, in turn, affect metabolism, organ systems, cellular processes and biochemistry. Indirect effects of the social environment (dashed arrows) can also occur via social effects on ecological factors (orange box). The combined direct and indirect effects of the social environment on physiological traits will lead to a range of selection outcomes (blue box). All processes and pathways take place within the context of abiotic factors.

One critical but often overlooked aspect of an animal's environment is social context. Nearly all animals are social at some point in their lives – typically during the most ecologically and evolutionarily consequential behaviours, including foraging, predator avoidance, migration and reproduction. Given that behavioural interactions and decision making are intertwined with physiology (Gilmour et al., 2005; Killen et al., 2013; Seebacher and Krause, 2017; Jolles et al., 2020; Debaere et al., 2024), then social context must be central to comparative and environmental physiology. Indeed, social dynamics influence physiological control systems such as endocrine signalling, redox balance and energy allocation, with downstream effects at the cellular level (Fig. 1). Environmental stressors can also destabilise social systems, resulting in feedback loops whereby environmental change alters social context (Kochhann et al., 2015; Fisher et al., 2021), which in turn may constrain physiological responses to further challenges (Gilmour et al., 2025).

In this Commentary, we discuss evidence for the various potential effects, both direct and indirect, of social behaviour on physiological traits. We then consider how these effects might affect measurements of these traits, and discuss their implications for the ability to cope with environmental change, including the capacity for evolutionary adaptation. We aim to illustrate why these effects, though documented in other fields, should be more routinely incorporated into physiological experimental design and interpretation. Indeed, the effects of social context on physiology have been explored in a subset of research studies for decades across multiple disciplines, from seminal work on social stress in primates (Sapolsky, 1990) to more recent studies in behavioural and ecological physiology (e.g. Creel et al., 2013; Dantzer and Newman, 2022; Brandl and Farine, 2024). Despite this foundation, however, these ideas have only gradually influenced comparative physiology more broadly.

Glossary **Additive genetic variance**

The portion of genetic variance attributable to the cumulative effects of individual alleles; it determines a trait's potential response to selection.

**Allostatic load**

The cumulative physiological burden imposed by chronic stressors, including social and environmental ones, leading to wear and tear on the body.

**Critical thermal maximum (CT_max_)**

The highest temperature at which an animal can maintain normal function before losing equilibrium, used to assess thermal tolerance.

**Heritability**

The proportion of observed variation in a trait due to genetic differences among individuals, measuring how much of the phenotypic variance can be attributed to genetic variance in a population.

**Indirect genetic effects (IGEs)**

When the genotype of one individual influences the phenotype of another through social interactions, thereby altering evolutionary dynamics.

**Phenotypic plasticity**

The ability of a single genotype to produce different phenotypes in response to environmental variation, including social environments.

**Reactive scope model**

A framework describing how organisms maintain physiological homeostasis, accounting for both normal fluctuations and overload caused by chronic stress.

**Social buffering**

A reduction in physiological stress responses due to the presence or interaction with conspecifics, especially familiar or affiliative individuals.

**Social stress**

Physiological stress resulting from social interactions such as aggression, competition or instability in dominance hierarchies.

## Social context is often overlooked in physiological research

Consideration of social context has a central role in other fields of biological research (e.g. Webber et al., 2023; Svensson and Sheldon, 1998; Rodrigues, 2018; Bro-Jørgensen et al., 2019; MacLeod et al., 2023), and the proximate influence of the social environment has long been recognised in behavioural and physiological ecology (e.g. Sapolsky, 1990; Creel et al., 2013). However, although many studies have revealed how social context affects endocrine, metabolic and stress responses, these insights have not been consistently accounted for in physiology in terms of experimental design, data interpretation or extrapolation of findings to natural systems (see Fig. S1 and Supplementary Materials and Methods).

Comparative physiology has traditionally emphasised abiotic factors such as temperature and oxygen, with social variables often treated as secondary or as uncontrolled ‘noise’. As the field increasingly transitions toward the realm of ecophysiology, there is a growing opportunity and need to more consistently integrate social context as a fundamental environmental dimension. Standard physiology textbooks devote entire chapters to abiotic factors such as temperature or oxygen but rarely acknowledge the physiological consequences of an animal's social surroundings, even though social dynamics alter hormone levels, energetic demands and stress responses. Moreover, social effects can interact with abiotic stressors (MacLeod et al., 2023), producing synergistic, additive or antagonistic outcomes (Munson et al., 2025; Tiddy et al., 2024).

Even as efforts to integrate physiology with behaviour have increased (Gilmour et al., 2005; Killen et al., 2013; Debaere et al., 2024), studies have typically centred on traits such as foraging, locomotion or predator avoidance, often without accounting for the social environment in which these behaviours are expressed. A major barrier is the inherent complexity and variability of social effects, which are more difficult to quantify and standardise than many abiotic factors. For the potential influence of temperature and oxygen availability, general principles have been established (e.g. Angilletta, 2009), rooted in shared physical and biochemical processes across taxa. In contrast, social effects are often more variable within and among species, posing a challenge for standardisation. For many species, we lack even a basic understanding of what constitutes a ‘normal’ social environment.

Overlooking social context in comparative physiology can distort trait values, reduce experimental repeatability and produce misleading ecological or evolutionary conclusions (Table 1; discussed further below). Importantly, social context not only influences physiological outcomes but also affects how we measure them. Experimental setups that overlook naturalistic social conditions might inadvertently introduce artefacts. Thus, we propose that physiologists should account for social environments as rigorously as they do for abiotic variables.

**Table 1.** Consequences of overlooking versus integrating social context in studies of comparative physiology

Dimension	Risk of overlooking social context	Benefit of integrating social context	Example of affected measure or scenario
Trait measurement accuracy	Physiological values might be distorted by social isolation or stress artifacts	Measurements reflect natural states and social modulation of physiology	Measuring resting metabolic rate or cortisol levels in animals housed alone
Interpretation of plasticity	Misses socially induced variability; might underestimate flexibility or context dependence	Captures plastic responses triggered by social status, competition or group dynamics	Assessing thermal tolerance without accounting for social stress during acclimation
Experimental repeatability	Variability due to uncontrolled or undocumented social conditions across studies	More reproducible results through standardised social setups	Comparing hormone levels across labs that house animals under different social densities
Ecological relevance	Lab results might not generalise to natural social settings	Findings more accurately reflect physiological function in real-world group contexts	Studying predator response in solitary animals versus in groups
Evolutionary inference	Might mis-estimate heritability or selection gradients if social interactions influence traits	Better insight into evolutionary potential and genotype-by-environment interactions	Inferring selection on stress responses or growth rates without accounting for social dominance
Stress and welfare effects	Higher stress or poor welfare can confound physiological outcomes	Reduced baseline stress through social buffering improves data quality	Testing immune function in isolated versus pair- or group-housed animals
Experimental design flexibility	Simplifies control but excludes a major environmental factor	Enables hypothesis-driven testing of social effects and interactions with other variables	Designing factorial experiments that include group size or social familiarity as factors
Conservation and applied insights	Overlooks social constraints on coping mechanisms or adaptive capacity	Informs on how social structure affects resilience under environmental change	Predicting range shifts or habitat use without factoring in group cohesion
Comparative interpretations	Overgeneralises across species with differing social systems	Enables trait comparisons that consider social context as an ecological and evolutionary variable	Comparing hormonal or metabolic traits between solitary and group-living species

## Social effects affect trait measurement

We believe that overlooking social context in comparative physiology studies is likely to create systematic biases that compromise the accuracy and comparability of physiological measurements. Although most experimental work to date has concentrated on how social context modulates endocrine profiles and whole-animal metabolic rate, these effects almost certainly cascade to organ, tissue and cellular processes in ways we have only begun to quantify (Fig. 1). Hormones and neurotransmitters sit atop hierarchical control networks, and once perturbed by social context, their downstream targets span every physiological system (Table 2). Elevated glucocorticoids, for example, re-allocate glucose and lipid utilisation, modulate immune cell expression and activity, alter cardiac output, blunt reproductive-axis signalling and can affect neural plasticity. Likewise, chronic shifts in metabolic rate might link with mitochondrial density, oxidative balance and metabolite availability, possibly influencing thermal limits, hypoxia tolerance, growth efficiency and even sensory acuity. Because these endocrine and metabolic ‘master regulators’ often operate through shared molecular pathways, any trait – be it haematocrit, heat shock-protein expression, antioxidant capacity or gut barrier integrity – might already be affected by an animal's social environment before the investigator applies an experimental treatment.

**Table 2.** Potential effects of social environments on different physiological systems and mechanisms

Physiological system/mechanism	Socially mediated effects	Potential mechanisms or pathways	Example references
Cardiorespiratory	Altered heart rate, ventilation rate, oxygen uptake, aerobic capacity	Social stress or buffering; group position affecting oxygen demand	Mignucci et al. (2021); Thomas and Gilmour (2012); Wascher et al. (2008)
Nervous	Changes in brain size, neurogenesis, neural investment	Social enrichment or deprivation; altered neurotrophic signalling	Branchi et al. (2006); Fischer et al. (2015)
Endocrine	Variation in cortisol, oxytocin, growth hormone, sex steroids	Social dominance, affiliation and stress buffering	Kikusui et al. (2006); Culbert et al. (2019); LaDue et al. (2023)
Immune	Immune suppression or enhancement	Stress hormone signalling; epigenetic effects of social experience	Gleeson (2007); Laubach et al. (2021)
Digestive/nutritional	Changes in feeding rate, nutrient uptake or diet composition	Competition, facilitation, social learning of food sources	Filby et al. (2010); Rosenfeld et al. (2015); Zhang et al. (2018)
Gut–brain axis	Altered gut microbiome	Microbiota modulation of HPA axis and corticotropin-releasing hormone neurons	Soares et al. (2019); Wu et al. (2021)
Metabolic rate	Elevated or suppressed standard or active metabolic rate	Isolation stress, social buffering, group size effects	Nadler et al. (2016); Killen et al. (2014); Zhang et al. (2025)
Locomotor function	Shifts in movement efficiency, burst speed or endurance	Group dynamics; social pressure; spatial position in groups	Marras et al. (2015); Killen et al. (2017); Brandl et al. (2025); Tiddy et al. (2025)
Muscle physiology	Muscle damage, performance declines or hypertrophy	Aggression, social stress, reproductive displays	Beaulieu et al. (2014); Fialkowski et al. (2021)
Mitochondrial function	Changes in density, efficiency or thermal tolerance	Group huddling; developmental or acute plasticity caused by changes in activity patterns	Zhang et al. (2018); Gilbert et al. (2010)
Protein/enzyme expression	Heat shock protein or enzyme expression shifts	Social stress-induced cellular responses	Currie et al. (2010); Pohorecky et al. (2004)
Cellular membranes	Changes in membrane fluidity or oxidative damage	Reactive oxygen species from social stress; altered thermal regimes chosen by social group changes membrane fluidity; altered fatty acid intake	Mougeot et al. (2009); Killen and Brown (2006)
Osmoregulation	Altered ion balance or stress-recovery ability	Hormone-linked osmoregulatory disruption; increased blood flow through gills due to social stress increases ion loss	Katayama et al. (2018)

For each system, we provide examples of socially mediated effects, possible mechanisms or pathways, and supporting literature. HPA, hypothalamic–pituitary–adrenal.

### Effects of social presence on trait measurements

For gregarious species, social grouping can dampen the primary stress response by suppressing hypothalamic–pituitary–adrenal (HPA) axis activity and accelerating recovery from stress (Kikusui et al., 2006; Culbert et al., 2019; Kiyokawa et al., 2014). Recognition of familiar companions through sensory and neural pathways also modulates endocrine signalling. Social interaction can also stimulate oxytocin release, which not only inhibits cortisol secretion but also promotes social affiliation and reduces negative emotional responses (Neumann et al., 2000; Kikusui et al., 2006). The neural circuits underlying these effects are well characterised, with oxytocin-expressing neurons in the paraventricular nucleus serving as key integrators of social sensory information (Menon and Neumann, 2023). These neurons are activated by various social stimuli and link to brain regions involved in stress regulation, reward processing and social memory formation, illustrating the interconnectedness of these systems.

The metabolic consequences of isolation versus group housing are equally profound. For some social species, living in groups is associated with lower metabolic demand, probably due to reduced individual vigilance (Roberts, 1996). Often referred to as the ‘calming effect’, the presence of conspecifics can reduce overt signs of distress, and individuals previously exposed to conspecifics might maintain lower resting metabolic rates even when measured alone (Chrétien et al., 2021). Visual and olfactory cues from conspecifics can reduce metabolic rate in social fish, suggesting a stress-buffering role of shoaling (Nadler et al., 2016). Grouping can also reduce anxiety-like behaviours and alter metabolic rate and resource allocation (Zhang et al., 2025), and fish housed in groups exhibit lower resting heart rates than when isolated in respirometers (Mignucci et al., 2021). However, social effects on metabolic demand may be nuanced, as greylag geese (*Anser anser*) show varying heart rate responses to social interactions, depending on whether family members or non-affiliated individuals are involved (Wascher et al., 2008).

### Effects of social stress on trait measurements

The physiological consequences of social stress (see Glossary) have been predominantly studied in relation to dominance hierarchies, especially in pairs of dominant–subordinate animals (Sloman and Armstrong, 2002), and are generally more extreme for subordinate individuals. We use the term ‘social stress’ here in its widely adopted sense while recognising that recent work (e.g. Gilmour et al., 2025) encourages a more mechanistic framing under the reactive scope model (see Glossary; Romero et al., 2009), which clarifies that it is not the social behaviour itself that is damaging, but the accumulation of physiological wear and tear when physiological mediators remain chronically within reactive ranges (Gilmour et al., 2025). Indeed, although the stress response is necessary for homeostasis in the face of acute stressors, prolonged activation can impair physiological function and influence an organism's response to additional stressors, and have adverse effects on growth, reproduction and immune function (e.g. Gleeson, 2007; Ambrée et al., 2018). In some species, subordinates within social hierarchies show an increase in metabolic rate that is related to the amount of aggression they receive (Sloman et al., 2000; Killen et al., 2014). Chronic cortisol elevation associated with subordinate social status during agonistic encounters has been proposed as one of the mechanisms contributing to this effect, and can be modulated by group size, aggression rates and stability of social relationships (Sapolsky, 1990; Pickering and Pottinger, 1995; Sloman and Armstrong, 2002; Bessa et al., 2021). Thus, from a methodological perspective, dominance-related differences in baseline physiology mean that studies sampling from populations without controlling for social status may inadvertently introduce measurement variance or bias.

The relationship between social status and stress hormones varies considerably among species and environmental conditions, with some studies finding higher glucocorticoid levels in dominants and others in subordinates (Creel et al., 2013). In some species, maintaining social dominance can be costly, particularly when hierarchies are unstable or rely on aggression, such as in some cichlid species (Fialkowski et al., 2021). In species that are territorial, the cost can increase with territorial value (e.g. dwarf cichlids, *Apistogramma* spp.: Kochhann and Val, 2017). A commonly reported cost across taxa is oxidative stress, often linked to upregulation of the reproductive axis in dominant animals (e.g. mammal: Beaulieu et al., 2014; fish: Fialkowski et al., 2021). During social ascent, dominant individuals may show increased aggression, courtship behaviour, gonadal growth and secondary sexual coloration – factors that elevate plasma reactive oxygen species (ROS), markers of oxidative damage. Concurrent investment in colourful traits may divert carotenoids away from systemic antioxidant defences (e.g. red grouse, *Lagopus scotica*: Mougeot et al., 2009). This imbalance between ROS production and antioxidant capacity results in oxidative stress, which can impair physiological function and reduce fitness. These costs may be transient or sex biased, depending on the stability of dominance hierarchies or seasonal reproductive effort, as demonstrated in mammals and birds (Beaulieu et al., 2014; Cram et al., 2015).

Social context can also modulate physiological stress and endocrine responses associated with the intensity or duration of reproductive states. For example, in male Asian elephants (*Elephas maximus*), musth and associated heightened androgen levels last longer in the presence of other males, but this response is briefer in individuals with low body condition (LaDue et al., 2023). In mandrills (*Mandrillus sphinx*), the mating season corresponds with increases in oxidative stress in females regardless of rank, but only in dominant males (Beaulieu et al., 2014). Similarly, in house mice (*Mus musculus domesticus*), the combination of reproductive effort and social competition increases oxidative damage (Garratt et al., 2012).

### Indirect pathways of social bias in measurements

Social interactions may also indirectly influence an animal's acute physiological state by altering food intake. For instance, real or perceived competition in social settings can stimulate feeding behaviour, increasing intake in anticipation of resource scarcity. Social foraging can also enhance access to food by increasing the likelihood of encountering food items, but in resource-limited environments, it may lead to reduced intake as a result of competition or resource depletion (Galef and Giraldeau, 2001). In hierarchically structured groups, subordinate individuals often face restricted access to food – either through direct exclusion or by self-restricting to avoid antagonism – resulting in reduced or lower-quality food intake (Cutts et al., 1998; Wong et al., 2008; Filby et al., 2010). Social influences can also affect food preference through mechanisms such as social learning, where individuals adopt the food choices of conspecifics, even when those choices are suboptimal (Galef and Whiskin, 2008; Jolles et al., 2011).

These socially driven changes in both the quantity and quality of food consumed can affect physiological systems. Altered food intake modulates endocrine status (Bronson and Heideman, 1990) and influences metabolic traits, energetic costs of digestion (Secor, 2009; Killen, 2014), the accumulation and composition of body lipids and essential fatty acids (Killen and Brown, 2006; Killen et al., 2007), and the morphology and functioning of the gut (Stojanović et al., 2022; Fernandes et al., 2024). Such changes, in turn, may have implications for energy allocation, growth, immune function, gut plasticity and thermal tolerance. Thus, social effects on feeding behaviour, even during routine holding in the laboratory, represent an important and often underappreciated pathway through which the social environment shapes physiological state and performance.

Emerging evidence also suggests that social–physiological interactions may involve bidirectional feedback loops with the gut microbiome. In addition to direct transfer of microbiota among social group members (Archie and Tung, 2015), gut microbial composition can also be altered by social stress through changes in gut permeability and immune responses (Delaroque et al., 2021). In turn, microbiota can have a range of effects on individual stress responses through the influence of bacteria-derived hormones and metabolites (Sandhu et al., 2017), and can feed back to modulate social behaviour through stress-response pathways in the brain, with specific bacterial species influencing corticosterone levels during social encounters (Wu et al., 2021).

Group dynamics can also change the energetic costs of locomotion. In terrestrial birds, for example, leadership attempts during group movements significantly increase heart rate compared with solitary movement at equivalent speeds (Brandl et al., 2025), and in schooling fishes, hydrodynamic factors caused by movement relative to neighbours reduce both aerobic and anaerobic energy use while swimming in groups (Marras et al., 2015; Zhang et al., 2024). However, most performance assays typically measure animals in isolation. If individuals of social species are tested singly, we may over-estimate the oxygen needed to migrate, disperse or escape predators. Although it has not been thoroughly investigated, this source of error is likely to be compounded under environmental stressors. When temperatures rise or oxygen falls, an individual's aerobic scope for movement can be reduced but, in a group such as a fish school, hydrodynamic subsidies may mitigate this, possibly allowing fish to maintain swimming speed and, by extension, range and foraging success. Modelling climate-driven range shifts or thermal or hypoxic ‘dead zones’ with single-fish data could thus predict local extinction, whereas, in reality, social movement could facilitate persistence.

### Developmental and transgenerational sources of measurement bias

**Fig. 2.**
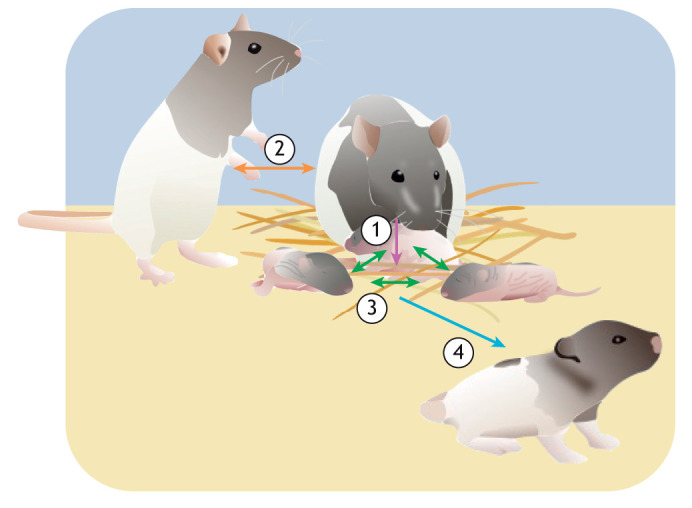
**Developmental and intergenerational social effects on physiological traits.** Parents can affect their offspring (1, purple arrow) either through the quality of their care or by genetic mechanisms. Additionally, the parent's social environment (2, orange arrow), including their position within a hierarchy, can also influence offspring physiology. Interactions among litter mates (3, green arrows) are an important part of the early developmental environment that can influence how individuals respond to later environments. Both within- and across-generation social effects can also affect physiological plasticity (4, blue arrow), such that differences in parental care and early social environment can result in genetically similar individuals displaying divergent phenotypes (e.g. size).

During development, the phenotype of organisms, including their physiology, is highly plastic and may change considerably in response to environmental conditions (Fig. 2). These changes can have lasting effects into adulthood and across generations (Burton and Metcalfe, 2014), through genetic or epigenetic mechanisms (Keller, 2009; Schneider et al., 2017; Laubach et al., 2021; Szyf, 2011). Social environment effects may be especially influential during sensitive developmental windows (Snell-Rood, 2013; Ruthsatz et al., 2020), with long-term consequences for physiological traits.

Social factors such as conspecific density, group size and social position can all shape individual physiology. Early social environments influence immune function (Baxter et al., 2021), stress reactivity and growth (Thünken et al., 2016). In zebrafish, isolation-induced behavioural changes are linked to altered brain activity in regions involved in social behaviour, stress and sensory processing (Tunbak et al., 2020). High conspecific density can promote alternative physiological phenotypes; for instance, brown trout (*Salmo trutta*) raised in high-density environments are more likely to develop migratory morphs, whereas low-density conditions favour residents (Olsson et al., 2006). In some species, density during early development even affects sex determination (Davey and Jellyman, 2005), and group size during early life influences neuroendocrine function, brain development (Fischer et al., 2015) and nerve growth factor levels (Branchi et al., 2006), with downstream effects on metabolism and immune function (Nyman et al., 2020).

Social effects on physiology can span generations through non-genetic inheritance, often through parental care. In species with parental provisioning, the absence of one or both parents can elevate offspring stress (Pillay and Rymer, 2021; Antunes et al., 2021; Damián et al., 2018). In rats, maternal licking and grooming influence offspring stress sensitivity, sociality and care-giving behaviour (Francis et al., 1999; Parent and Meaney, 2008; Perkeybile and Bales, 2017), and increased maternal care causes epigenetic modifications in genes linked to immunity and ageing of offspring in spotted hyenas (Laubach et al., 2021). Parental social conditions also matter: dominant male mice sire sons that show faster early-life growth and distinct liver gene expression profiles compared with sons of subordinate males, suggesting that paternal social status can shape offspring metabolism and growth-related pathways through non-genetic inheritance (Cauceglia et al., 2020), and socially enriched female rats produce heavier offspring with enhanced cortical plasticity (Sparling et al., 2010; Faraji et al., 2018). Maternal stress, infection or malnutrition can impair care and affect offspring brain development and cognition (Fitzgerald et al., 2020; Pittet et al., 2019; Bredy et al., 2004).

Group composition during development adds further complexity. Individuals raised with familiar kin often grow faster, possibly as a result of reduced aggression and the experience of more-stable hierarchies (Thünken et al., 2016). Moreover, group position can strongly shape physiology. In birds, asynchronous hatching gives older nestlings a growth advantage (Stoleson and Beissinger, 1997), whereas in matrotrophic fish, earlier-born offspring are larger (Schrader and Travis, 2012). In great tits, smaller broods show heightened stress responses, and brood sex ratio influences physiology-linked personality traits (Naguib et al., 2011).

## Social context alters environmental tolerance

The social environment not only modifies physiology but can also alter responses to temperature extremes, hypoxia and other environmental challenges. Therefore, tolerance limits measured without considering social dynamics may not predict actual performance in nature, where animals experience stressors within their normal social context, as we discuss in detail in this section.

### Social stress compounds effects of environmental stressors

Social stress can exacerbate the effects of other physiological stressors. Prolonged elevation of cortisol and other mediators can narrow an individual's reactive scope (Gilmour et al., 2025), increasing vulnerability to increased allostatic load (see Glossary). In fishes, elevated metabolic rates in subordinates can impair their ability to cope with hypoxia as a result of increased oxygen demand (Thomas and Gilmour, 2012), and subordinates might also exhibit reduced thermal tolerance (Leblanc et al., 2011). Bard et al. (2021) found that critical thermal maximum (CT_max_; see Glossary) is lower in subordinate individuals and remains suppressed after recovery when cortisol is administered, highlighting the role of cortisol in modulating thermal limits. In some bird species, dominant individuals can cause feather thinning in subordinates through pecking, reducing insulation and increasing susceptibility to thermal stress (Vestergaard et al., 1993). In species where dominance is energetically costly, dominant individuals might be more prone to mass loss or starvation during food shortages. For instance, in red deer, subordinates have lower heart rates and energy demands and show less mass loss under restricted feeding, potentially providing an advantage during winter (Turbill et al., 2013).

### Social buffering enhances stress tolerance

Although group living can sometimes induce stress, the calming effect of conspecifics in social species can also buffer physiological responses to external stressors by influencing metabolic, endocrine, neurological and immune systems (Gust et al., 1994; Kikusui et al., 2006; Kiyokawa and Hennessy, 2018; Yusishen et al., 2020; Pintos et al., 2024). The intensity of the effects of social stress versus social buffering (see Glossary) may vary with the degree of social isolation or social competition experienced. Bailey and Moore (2018) propose that an ‘index of social isolation’, describing the degree of mismatch between optimal and actual social interaction, could help in estimating the magnitude of physiological responses to social environments and their interactions with other environmental factors.

Group living can buffer physiological stress through a range of behavioural and physiological mechanisms. For example, individuals can reduce their stress responses by copying the behaviour of parents or calm conspecifics (Kikusui et al., 2006). In heated honey bees (*Apis mellifera*), individual workers first fan at ∼48°C, whereas groups of ten begin near 39°C and do so twice as often, effectively expanding the colony's thermal safety margin (Cook and Breed, 2013). In lake sturgeon (*Acipenser fulvescens*), the presence of conspecifics lowers both cortisol levels and cellular responses to thermal stress (Yusishen et al., 2020), and in Italian riffle dace (*Telestes muticellus*) exposed to flowing water, group living reduces cortisol levels and oxidative stress while enhancing antioxidant defences, enabling better swimming performance under challenging hydrodynamic conditions (Schumann et al., 2023). In huddling species, group living reduces the metabolic cost of thermoregulation and can enhance mitochondrial density (Gilbert et al., 2010; Zhang et al., 2018; Wang et al., 2020). Similarly, hypoxia tolerance in *Drosophila* improves with group size, probably as a result of group size-dependent reductions in metabolic rate (Burggren et al., 2017).

This stress-buffering role of social behaviour is especially relevant when considering a species' capacity to withstand environmental change. However, the effectiveness of social buffering depends on factors such as partner familiarity, social relationships and group size. For example, social buffering occurs with both familiar and unfamiliar individuals, but tends to be stronger with familiar partners, whereas aggressive interactions can diminish its benefits (Hennessy et al., 2008; Kiyokawa and Hennessy, 2018). Group size also influences the effectiveness of huddling and the neuroendocrine response to stress (Gilbert et al., 2010; Kikusui et al., 2006). If environmental change disrupts these social factors – perhaps by reducing group size in gregarious species under warming conditions – then the ability to buffer stress might also vary across environments.

### Social modulation of behavioural responses to environmental stress

It is often assumed that animals select habitats that optimise energy balance (Borowiec et al., 2018; Tucker and Suski, 2019). In social species, however, space use is influenced not just by environmental conditions but also by the behaviour and presence of conspecifics. Individuals might choose to remain under suboptimal conditions, such as hypoxia, acidification or thermal stress, if social partners are present, highlighting a trade-off between social affiliation and physiological stress (Currie and Tattersall, 2018; Tucker and Suski, 2019). In some cases, dominant individuals control access to optimal habitats (Alanärä et al., 2001), whereas in others, social cues override individual preferences, such as in sticklebacks that choose cooler habitats when a shoal is present, even if warmer environments are preferred in isolation (Cooper et al., 2018). Thus, if an investigator measures an animal's ‘preferred habitat’ in isolation, or without considering social dynamics, the resulting estimate may bear little resemblance to what that same animal would occupy in nature. Under such circumstances, we risk overestimating or underestimating critical avoidance thresholds for temperature, oxygen or pH, and hence mis-predicting how populations will redistribute under environmental change. Air-breathing fishes provide a compelling example of how social context can override individual physiological demands. Recent studies have shown that group-level effects on activity, such as aggression from dominant individuals, strongly influence the tendency to surface for air, potentially decoupling this behaviour from maintenance oxygen demands or stressors such as hypoxia (Killen et al., 2017; Pineda et al., 2020).

### Understanding climate responses requires consideration of social context

At landscape scales, the same social forces that influence physiology also affect where animals live. Many animals use social information to guide habitat selection and migration (Mueller et al., 2013; Whitehead et al., 2004), and these cues can either promote adaptive movement or lead to ecological traps (Sigaud et al., 2017). If we calibrate species-distribution models with ‘intrinsic’ thermal or oxygen limits measured in isolation, we ignore the fact that real-world movements are often determined by the actions of conspecifics. For example, traditions inherited across generations can anchor groups to formerly productive habitats, even after they have become non-optimal (Keith and Bull, 2017; Barrett et al., 2019), whereas horizontal information flow among peers can catalyse rapid colonisation of novel areas when conditions shift. Individual variation in sociality further complicates predictions: bold, weakly social individuals often disperse more widely, but the bulk of the population may only follow once cohesive groups form (Cote and Clobert, 2007; Cote et al., 2011). Notably, physiological plasticity might compensate when social forces limit range shifts. For example, subordinate social status in fish can enhance thermal tolerance through upregulation of heat shock proteins (Currie et al., 2010; Sørensen and Loeschcke, 2007), and in the blue-tailed damselfly, social stress has facilitated poleward range expansions by improving cold tolerance (Lancaster et al., 2017; Wood et al., 2019). Environmental tolerance assays that omit social dynamics might therefore mis-estimate not only when and where animals want to move but also whether they are capable of surviving once they get there.

## Social dynamics affect evolutionary predictions

A core aim of comparative physiology is to understand evolutionary influences on physiological systems and to predict how these systems will respond to environmental pressures (Prosser, 1950). This often involves a specific focus on mechanisms and their apparent adaptive functions, without explicit acknowledgement that the form and regulation of a given organ, tissue, transmitter, hormone or receptor might have been influenced by selection pressures arising from the social environment, as we consider below.

### Social constraints on independent trait evolution

Social environments can create evolutionary constraints by linking physiological systems that serve multiple functions, preventing independent optimisation of different traits. When the same physiological mechanisms regulate both social behaviour and environmental tolerance, selection on one function necessarily affects the other. For example, the anabolic effects of testosterone on muscle tissue benefit physical confrontations and territorial displays, but force energetic trade-offs with immune function, constraining species' ability to simultaneously optimise both social competition and disease resistance (Trumble et al., 2016; Foo et al., 2017). Similarly, interferon-γ – a cytokine recognised for antiviral defence – has also been shown to modulate social behaviour, and reductions in this cytokine compromise both immunity and social interaction in domestic mice, again preventing independent optimisation of these functions (Filiano et al., 2016). The mudskipper vasotocin system exemplifies this type of constraint in an osmoregulatory context, as vasotocin simultaneously regulates osmoregulation and social behaviour through overlapping neural pathways (Katayama et al., 2018). This functional linkage means that evolutionary changes to improve osmoregulatory performance could inadvertently alter social competitive ability.

Such pleiotropies might become particularly important under environmental change. When selection pressures shift as a result of climate change or other stressors, adaptive responses in one domain could be prevented or compromised by linked functions in another. For example, if increased resource competition favours enhanced social dominance, selection for higher testosterone could compromise disease resistance precisely when environmental stress requires strong immune function. The result is evolutionary ‘stasis’, where optimal responses to environmental stressors are prevented by social constraints, or suboptimal compromises, where neither social nor physiological function is optimised.

### Indirect genetic effects and the social evolution of physiological traits

Social environments generate indirect genetic effects (IGEs; see Glossary), where the genotype of one individual influences the phenotype of another through behavioural interactions (Moore et al., 1997; Wolf et al., 1998). For physiological traits, this occurs when genetically based differences in social behaviour systematically affect the physiological performance of group mates. For example, individuals genetically predisposed to aggression might elevate stress hormones or reduce growth rates in subordinate group members (Brichette et al., 2001). These IGEs alter the total heritable variance available to selection by adding a social genetic component that can either enhance or oppose direct genetic effects (Bijma, 2010; Wolf et al., 1998), potentially accelerating or constraining evolution depending on whether the effects enhance or diminish trait expression in others (Santostefano et al., 2017). Even small IGEs can drive substantial evolutionary responses independent of changes in additive genetic effects (Moore et al., 1997). For comparative physiologists, this means that evolutionary predictions based solely on individual estimates of heritability (see Glossary) will be systematically biased, as they ignore the socially induced plasticity and genetic components that determine how traits evolve in group-living species. The effects of socially induced IGEs on physiological traits could be examined more widely, to fully understand potential responses to environmental change (Han et al., 2018).

### Effects on trait variability and heritability

The social environment can fundamentally shape evolutionary outcomes by altering trait variability, heritability and genetic architecture (Law, 2000; Crespel et al., 2024), meaning that estimates derived from isolated animals may misrepresent adaptive potential in natural settings. Social conditions induce plasticity within and across generations, influencing phenotypic variance, the strength and direction of selection, and the expression of additive genetic variance (see Glossary), thereby modifying heritability and the capacity for evolutionary change (Komdeur and Ma, 2021). For instance, social buffering could reduce variability in stress responses among genotypes, masking genetic differences from selection and constraining evolutionary change (Price et al., 2003; Wild and Traulsen, 2007). In contrast, genotype-by-environment interactions may shift selection depending on the prevailing social context (Nicolaus et al., 2016). Because heritability determines the rate of evolutionary change (Falconer and Mackay, 1996; Lynch and Walsh, 1998), any social influences on phenotypic variability or gene expression can alter evolutionary potential. In fishes, for example, genes involved in immune function and stress responses differ in expression with social density (Yarahmadi et al., 2016), and additive genetic variance for behaviour and growth potential also changes with social density (Mas-Muñoz et al., 2013; de Verdal et al., 2014; Crespel et al., 2021). Shifts in genetic correlations among traits, caused by social context, further shape evolutionary pathways through correlated selection (Teplitsky et al., 2014; Styga et al., 2019).

## Social context as both resource and confound: implications for experimental design

Although the effects of social dynamics on physiological traits present challenges, they also offer exciting opportunities for new perspectives on old physiological questions. Progress will come from three broad and complementary strategies: (1) integrating behavioural and social metrics into physiological study design; (2) leveraging technologies that allow group-level measurements; and (3) treating social context as a mechanistic variable rather than a confound. Within each of these broad approaches, more refined protocols can be developed to examine specific effects of the social environment on physiological traits. Emerging technologies for automated behavioural tracking (Nathan et al., 2022) and analytical tools for quantifying social interactions (Smith and Pinter-Wollman, 2021; Koger et al., 2023) offer powerful means of studying how physiology interacts with social context. Recent evidence even points to dedicated neural pathways for detecting social cues (Liu et al., 2025), suggesting that physiological responses to social environments may be more systematic and mechanistic than previously recognised. For extrapolation to natural ecosystems, frameworks such as the ‘spatial-social interface’ (Webber et al., 2023), which link social and spatial environments across scales, could also be extended to include physiological phenotypes.

Researchers should carefully consider how a species' natural social tendencies align with their experimental design. For instance, CT_max_ assays in fish are often conducted in groups to increase throughput (Raby et al., 2025), yet – depending on the species – this could trigger social buffering or socially mediated physiological activation, altering thermal tolerance outcomes. Conversely, when isolation is necessary for individual identification or environmental control, it should be used only where compatible with a species' sociality, or applied in less disruptive forms – such as permitting visual or olfactory contact. These issues are particularly important during early-life rearing, when inappropriate social conditions can induce long-lasting changes in stress physiology, metabolism, behaviour and environmental preference.

Even when group conditions are standardised, unmeasured social dynamics might still influence physiological and behavioural responses. Hierarchy formation, dominance interactions, social bonds and prior instability can simultaneously affect multiple physiological systems (Milewski et al., 2022), meaning that data collected during periods of social reorganisation might reflect transient rather than stable phenotypes. Allowing time for social stability to emerge before testing, and incorporating simple behavioural assessments (e.g. dominance, affiliative behaviour, responsiveness to isolation), can therefore improve the accuracy and interpretation of results.

To support implementation, we outline several practical steps applicable across taxa (Table 3). Standardising pre-experimental social history (e.g. familiarity, group size, sex ratio) can reduce variance, while aligning housing and handling with species-typical sociality (e.g. minimising isolation for social species, or providing social-buffering cues during acclimation) limits unnecessary physiological activation. Recording basic group-level social metrics enables these effects to be incorporated into analyses. Finally, although more demanding, IGEs could be quantified by manipulating social partner identity (e.g. pairing individuals with known genotypes or performance phenotypes) and partitioning variance using mixed-effects or animal models.

**Table 3.** Methodological artefacts and concerns that might arise when social context is not considered in physiological studies, and recommended means of mitigation

Scenario	Potential artefact/concern	Recommended mitigation
Isolated housing of social species	Elevated stress responses; inflated metabolic rates or hormone levels; developmental effects of isolation on traits; inhibited appetite; poorer condition	Use group housing when possible; allow acclimation to social conditions; validate isolation effects
Mixed dominance hierarchies without control	Individual variability in physiological traits due to social rank; asymmetries in food intake	Assess and account for dominance status
Unfamiliar group composition	Stress due to unfamiliarity; suppressed behaviour; altered metabolism and endocrine profiles	Use stable or familiar groups where possible; report group history
Variable group size or density	Confounding effects on metabolic rate, stress or growth	Standardise group size/density; report housing density in methods
Shared chambers (e.g. respirometry, swim tunnels)	Interference, increased activity, aggression, dominance artefacts; reduced locomotor costs in aquatic animals	Test individuals separately or use barriers; monitor for aggressive interactions
Lack of social cues during trials (e.g. testing alone)	Absence of social buffering; unrepresentative behaviour	Provide visual or olfactory cues; compare with group trials when feasible
Prior social experience not documented	Carryover effects on physiology or behaviour	Standardise and report rearing/social history; allow time for social stabilisation
Social effects confounded with treatment effects	Misinterpretation of treatment responses	Include social context as a factor in statistical models; factorial designs with social variables
Overlooking social structure in wild sampling	Sampling bias based on group position (dominant versus subordinate)	Stratify sampling by role or position; consider social metrics in field studies
Lack of species-specific social knowledge	Misapplied assumptions; stress or underperformance	Review natural history; consult behavioural literature; pilot tests to assess social sensitivity

## Conclusion

Work across behavioural ecology, neuroendocrinology and biomedical science has demonstrated that social context influences animal physiology. However, despite this body of evidence, these effects are incompletely integrated into comparative physiology's experimental and conceptual foundation.

The fields of comparative and environmental physiology have long aimed to uncover how organisms function and adapt to environmental change (Prosser, 1950). However, we risk two critical missteps. First, by routinely measuring animals in socially unrealistic or uncontrolled conditions, we might introduce unrepresentative measurements across physiological systems, from cells to whole organisms. Second, we then extrapolate these findings to natural populations, where trait expression is shaped by social dynamics that were absent or distorted in the experimental context. As a result, our current frameworks may be poorly equipped to predict ecological or evolutionary outcomes. Indeed, sociality itself might be a key mechanism by which animals buffer stress and adapt to environmental unpredictability (Komdeur and Ma, 2021), and understanding its physiological consequences requires recognition that social context is not noise – it is a key environmental variable with mechanistic and evolutionary importance for physiological trait expression.

To move the field forward, we believe that comparative physiologists should systematically consider social variables in experimental design and interpretation. This means choosing socially appropriate model systems, controlling or manipulating social conditions explicitly, or utilising technologies that allow physiological measurements in naturalistic group settings. Greater integration with behavioural ecology, evolutionary biology and quantitative genetics will also be essential. By closing the gap between how animals are studied and how they live, we can generate more accurate, ecologically relevant and predictive insights into organismal responses to environmental change.

## Supplementary Material

10.1242/jexbio.251374_sup1 Supplementary information
