# Supplementary material for: Further integrating social context into comparative and environmental physiology
Source: J Exp Biol. 2026 Feb 9;229(3):jeb251374. doi: 10.1242/jeb.251374 (PMC12951612; doi:10.1242/jeb.251374)
Supplement: Supplementary information [file jexbio-229-251374-s1.pdf]

Supplementary Materials and Methods

To examine the extent to which physiologists consider the social environment in their studies, we searched articles across all issues of the *Journal of Experimental Biology* to focus on vertebrates (Web of Science Core Collection, July 2025; animal* NOT insect* NOT drosophila NOT biomechanic* NOT mollusc* NOT isopod* NOT crustacean* NOT nematode* NOT arthropod* NOT invert* NOT cephalopod*). Using the returned articles, we then constructed a keyword co-occurrence network (using Web of Science's keywords plus, which integrates information using the R package Bibliometrix; Aria and Cuccurullo 2017) to identify general domains within comparative physiological research published in the *Journal of Experimental Biology*. We focused on *Journal of Experimental Biology* because it is a leading outlet for comparative and environmental physiology and therefore provides a representative analysis of research trends in the field. Focusing on one core journal also provides a cleaner indication of how frequently social context is explicitly incorporated within comparative physiology. Our intention was to illustrate patterns, rather than conduct an exhaustive bibliometric survey across journals.

Notably, the term *social* occurred in only 63 out of the 2,795 papers returned in this search (2.3%) and accounted for 73 out of 34,395 keywords plus in total (0.22%). It is striking that the word "social" or any derived term are entirely absent from the keywords plus clusters in this network (Figure 2). This is particularly surprising given the clear relevance of social behaviour to many of the processes represented. The omission of the social environment points to a persistent conceptual gap in the comparative physiology literature and suggests that many studies in ecophysiology approach animals as isolated units, despite increasing recognition that physiology and behaviour are deeply intertwined within social contexts (Milewski et al., 2022). This gap not only limits our mechanistic understanding but may also hinder our ability to predict organismal responses to environmental change in the real, socially structured world.

The complete list of the top 100 Keyword Plus phrases are as follows: 1. behaviour (268 occurrences); 2. locomotion (239); 3. metabolism (205); 4. evolution (191); 5. temperature (182); 6. fish (152); 7. energetics (143); 8. kinematics (128); 9. responses (124); 10.

Journal of Experimental Biology • Supplementary information

performance (113); 11. exercise (106); 12. flight (100); 13. skeletal-muscle (97); 14. speed (94); 15. thermoregulation (92); 16. birds (91); 17. stress (91); 18. oxygen-consumption (90); 19. hypoxia (89); 20. rainbow-trout (88); 21. oxidative stress (87); 22. muscle (85); 23. growth (82); 24. metabolic-rate (80); 25. vision (79); 26. mechanisms (73); 27. heart-rate (71); 28. mechanics (71); 29. orientation (70); 30. morphology (66); 31. patterns (66); 32. mitochondria (63); 33. animals (62); 34. mammals (59); 35. physiology (59); 36. climate-change (57); 37. expression (57); 38. hibernation (57); 39. cardiac function (53); 40. dynamics (51); 41. size (51); 42. swimming (51); 43. reproduction (50); 44. navigation (49); 45. ventilation (49); 46. neurons (47); 47. body-size (46); 48. trade-offs (46); 49. communication (45); 50. oxygen consumption (45); 51. phenotypic plasticity (45); 52. gene-expression (44); 53. model (44); 54. plasticity (44); 55. acclimation (43); 56. allometry (43); 57. gait (43); 58. system (43); 59. body-temperature (42); 60. ecology (42); 61. heart (41); 62. power output (41); 63. respiration (41); 64. water (41); 65. adaptation (40); 66. cost (39); 67. locomotion performance (39); 68. reptile (39); 69. transport (39); 70. bird (38); 71. terrestrial locomotion (38); 72. brain (37); 73. gas-exchange (37); 74. lizard (37); 75. sensitivity (37); 76. corticosterone (36); 77. discrimination (36); 78. echolocation (36); 79. Oncorhynchus mykiss (36); 80. oxygen (36); 81. diving (35); 82. diving behaviour (35); 83. migration (35); 84. walking (35); 85. feeding (34); 86. force (33); 87. hearing (33); 88. scaling (33); 89. sexual selection (33); 90. climate change (32); 91. energy-expenditure (32); 92. drag (31); 93. light (31); 94. movements (31); 95. ontogeny (31); 96. swimming performance (31); 97. temperature tolerance (31); 98. body temperature (30); 99. development (30); 100. marine mammals (30)

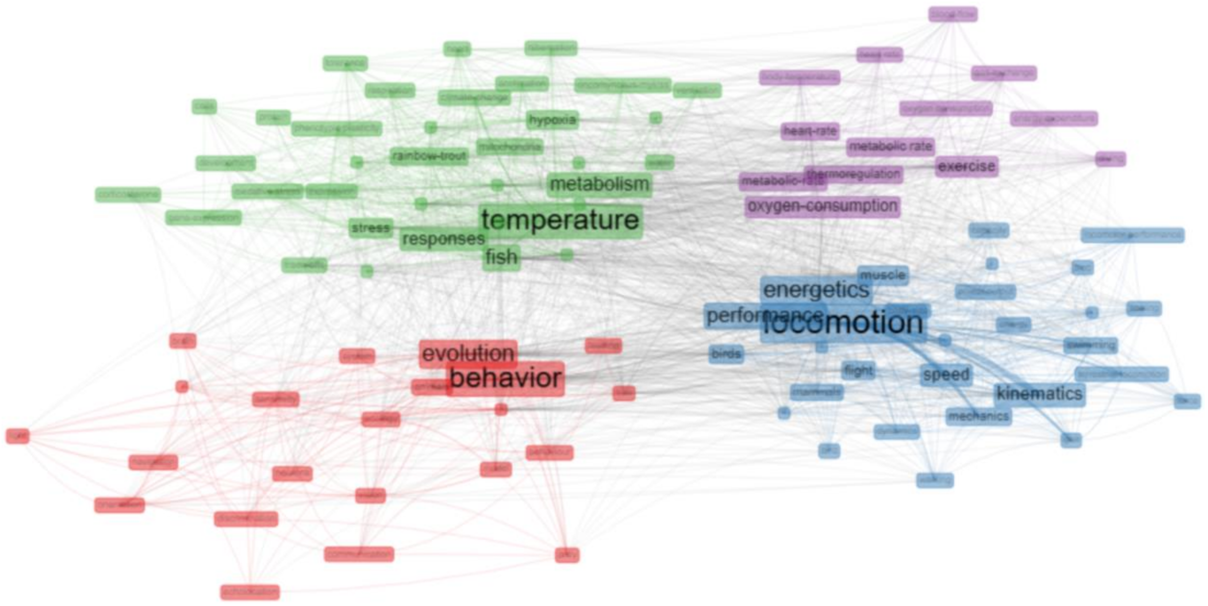

Fig. S1. Co-occurrence network of the top 100 Keywords plus from all articles published in the Journal of Experimental Biology, using Web of Science Core Collection. The keyword co-occurrence network (using the top 100 terms) across these articles forms several distinct clusters of topics, roughly focused on: (1) environmental stress physiology, particularly temperature, hypoxia, and associated oxidative responses (green); (2) metabolic and energetic processes, such as oxygen consumption, hibernation, and exercise physiology (purple); and (3) locomotor performance and biomechanics, including kinematics, muscle function, and swimming or flight (blue); and (4) behavioural mechanisms, including cognition, communication, and navigation (red).

LITERATURE CITED

Aria, M., Cuccurullo, C., D’Aniello, L., Misuraca, M. and Spano, M. (2024). Comparative science mapping: a novel conceptual structure analysis with metadata. *Scientometrics* **129**, 7055–7081.

Journal of Experimental Biology • Supplementary information
